# Understanding concerns after severe COVID-19: A self-imposed lockdown guarded by anxiety?

**DOI:** 10.1371/journal.pone.0287981

**Published:** 2023-07-19

**Authors:** Karin Törnbom, Alexandra Larsson, Katharina S. Sunnerhagen, Annie Palstam, Hanna C. Persson

**Affiliations:** 1 Institute of Neuroscience and Physiology, Rehabilitation Medicine, Sahlgrenska Academy, University of Gothenburg, Gothenburg, Sweden; 2 Department of Social Work, University of Gothenburg, Gothenburg, Sweden; 3 Department of Public Health and Community Medicine, Institute of Medicine, Sahlgrenska Academy, University of Gothenburg, Gothenburg, Sweden; 4 Department of Occupational and Physiotherapy, Sahlgrenska University Hospital, Gothenburg, Sweden; 5 Department of Neuroscience, Sahlgrenska University Hospital, Gothenburg, Sweden; 6 School of Health and Welfare, Dalarna University, Falun, Sweden; Stellenbosch University, SOUTH AFRICA

## Abstract

**Objective:**

Many people are struggling to get back to their lives after severe COVID-19. To facilitate their reintegration into everyday life, we need to understand how the process is experienced. We aimed to gain deeper knowledge about this process by interviewing persons one year after hospitalisation due to COVID-19.

**Methods:**

The study is based on a qualitative design, with eleven in-depth interviews conducted one year after discharge for COVID-19. Participants were recruited to form a heterogeneous sample with respect to age, gender and socioeconomic background. All interviews were analysed utilising inductive thematic analysis.

**Results:**

From the participants’ narratives four themes were identified: ‘Concerns and worries in everyday life’, ‘Supportive and concerned relatives’, ‘A new way of life–sorrows and advantages’ and ‘Seize the day–a greater awareness of one´s mortality’. Participants described how they tried to create a functioning everyday life. They were generally afraid of getting COVID-19 again and concerned about future life, where their lack of energy played a major role. Narratives were diverse regarding to what extent the struggle to cope was experienced as emotionally challenging or not.

**Conclusions:**

Participants described an unpredictable recovery after COVID-19, characterised by ups and downs, which created worries concerning their future. In some cases, the worry of getting COVID-19 again was strong enough to keep participants in their homes, as a self-imposed lockdown guarded by anxiety. However, the narratives also revealed gratitude towards being alive and having coped so well. This led to a more positive outlook on life with a greater focus on intrinsic values, close social relations and the deeper meaning of life.

## Introduction

According to early estimations, around 15–20% of patients positive for COVID-19 were hospitalised, and around 3–5% needed critical care [1, 2]. Fortunately, hospitalisations are decreasing with vaccinations and milder COVID-19 variants [3, 4]. However, the number of persons who have been hospitalised for COVID-19 at some point is high [5], and patients with a more severe initial infection seem to report long-term mental health problems more than others [6].

It is known that fatigue [7], anxiety, depression and other common mental health disorders are prevalent among COVID-19 patients [8], even 1-year after infection [9, 10]. Further, a recent study showed that severe COVID-19 increased the risk of long-term anxiety and depression, indicating a more negative impact on mental health for people with an initial severe infection [6]. Hospital treatment in intensive care units (ICUs) was shown to increase the risk of developing depression, anxiety and other symptoms related to post-traumatic stress disorder (PTSD) [11]. Moreover, COVID-19 patients with mental health disorders had a higher risk of mortality two years after ICU discharge [8, 12].

Anxiety and distress are common human responses to a perceived threat or an unpleasant event [13]. Commonly, anxiety is a feeling people can overcome when the threat is no longer imminent, but it can also continue at an abnormally high level that is difficult to cope with [13]. The current pandemic was characterised by inconsistent communication in the media, which led to misunderstandings about the rules and recommendations that were imposed [14]. It has been argued that this ambiguity about which recommendations were “real” together with an overall uncertainty about the future created a huge emotional challenge for many people [14–16].

For people who have survived severe COVID-19, it may be even more difficult to distinguish a real threat of infection from an overall pervasive feeling of fear. However, when the risk of infection is low, it is important to counteract these negative spirals of anxiety and try to resume activities that provide pleasure [14, 17]. Close social support was found to be important for alleviating worry about COVID-19 [15, 18], and therefore it has been argued that helping and supporting the entire family is beneficial [15].

Persisting symptoms after COVID-19 may be experienced for a long and unknown amount of time, and limited evidence exists about how to manage some symptoms [17]. This uncertainty, coupled with an exaggerated anxiety about the future, leaves people in a precarious and unknown situation [19]. It has been argued that these changes can cause an identity crisis that is particularly challenging and even a lost sense of who one is [20].

Considering the risk for long-term consequences, it is crucial to investigate how people experience and deal with life in a later phase, after hospitalisation for COVID-19. The purpose of this study was to explore life experiences at 1-year after hospitalisation for COVID-19 in Sweden, regarding mental health and social and emotional aspects.

## Methods

### Study design

This is a qualitative study with an explorative approach, comprising individual interviews with persons conducted one year after hospitalisation for COVID-19. All interviews were analysed with inductive thematic analysis with reference to a realist paradigm. The purpose of the study was thoroughly communicated to all participants before the study started and written informed consent was collected prior to the interviews. The study adhered to the consolidated criteria for reporting qualitative research (COREQ) guidelines [21] was approved by the Swedish Ethical Review Authority (Dnr: 2020–03046, 2020–0392) and followed the Helsinki declaration. The interview guide, and results were discussed in cooperation with two patient partners with lived experiences of COVID-19, one having been hospitalized due to COVID-19 and one having had long COVID-19 without needing inpatient care.

### Sampling and participants

A purposive sample of participants was recruited from the cohort Life in the Time of COVID study in Gothenburg (GOT-LOCO). The GOT-LOCO cohort included persons previously hospitalised due to COVID-19 (July 2020 to February 2021) in Västra Götaland Region, Sweden. Patients were enrolled in the GOT-LOCO while still in hospital and included if they were non-contagious, had an expected hospital care duration of over five days, were 18 years or older and had lived in their own housing prior to hospitalisation [22]. Patients who were unable to provide informed consent, had an expected one-year mortality prognosis or were not Swedish residents were excluded from the GOT-LOCO study.

In accordance with the study aim, participants from the GOT-LOCO cohort were selected for interview when approximately twelve months had passed since their discharge. In order to achieve a large variation in experiences, eligible participants were selected based on differences in the following characteristics: age 18–65 years old (working age), sex, educational level, level of care in the acute setting, number of days in hospital and symptoms reported at the three month follow-up [8].

According to the inclusion criteria, 14 persons were purposively selected to form a sample characterised by a high level of variation in personal characteristics. This group was contacted by an invitation letter followed by a phone call a few days later. A total of 12 persons agreed to participate, among whom one later declined. Thus, 11 persons (9 men and 2 women) finally chose to participate. The mean age was 54 years (42–62) at the time of COVID-19 infection, seven participants had received intensive care at their acute illness and four had been an inpatient in another ward unit. Four participants worked full-time, while seven were on full or part-time sick leave. Six participants had a university degree, and five had high school as their highest degree. The interviews were held at approximately 1-year after discharge for COVID-19. All interviews were held in Swedish.

### Data collection

Due to the risk of transmission of COVID-19, it was considered important to let participants decide whether or not they felt safe to undergo face-to-face interviews. Upon request, two participants chose face-to face interviews, five chose to be interviewed by phone, and four chose digital zoom interviews. All interviews were individual and conducted by the first author (KT). KT is a woman, has a PhD in medicine, is a social scientist, has previous experience in performing qualitative studies but had no previous contact with the participants. The original interview guide was revised as a semi-structured interview guide with open-ended questions after discussions among all authors. Interviews began with questions about demographic background and continued with open-ended questions that were focused on the purpose of the study.

Participants were encouraged to speak freely about each topic, and therefore the material obtained was multifaceted with rich content. During the course of the interview phase, a few questions were added to cover aspects that needed more attention. The interview guide appears in its complete form in both English and Swedish (**S1** and **S2 Files**).

All interviews (n = 11) were held between February 7–21, 2022 with a duration ranging between 33–73 minutes. After each interview, written notes were made with reflections about the contents.

### Data analysis

The interview material was transcribed verbatim and analysed with inductive thematic analysis following guidelines constructed by Braun and Clarke [23]. Thematic analysis is a commonly used method for identifying, analysing and reporting themes in qualitative interviews. It is also used as a tool to interpret various aspects and enables an in-depth exploration of the participants’ experiences without any pre-existing theoretical framework. Themes presented are therefore an unaltered representation of participants’ statements and narratives, providing a deeper understanding of their lived experiences.

All interviews were read and re-read by the first and second authors (KT and AL) to obtain a sense of the whole. In the next phase codes that were relevant for the research questions were marked with colour codes by both authors separately. Then, discussions were held to identify themes and potential sub-themes. The next phase involved refining themes and sub-themes, and in this phase some themes were aggregated while minor themes with less substance were removed due to inconclusiveness. Both authors (KT and AL) paid particular attention to how the parts of the text were related to the whole and switched their focus between these two aspects throughout the analysis process. This procedure was important to ensure the validity of the themes in relation to the material in its entirety. The contents of each theme were re-read to ensure that they were not overlapping. In the last stage, analysis and results were discussed again among all authors, ensuring the validity of themes in relation to the dataset.

## Results

Through the inductive thematic analysis used, four themes were identified: ‘Concerns and worries in everyday life’, ‘Supportive and concerned relatives’, ‘A new way of life–sorrows and advantages’ and ‘Seize the day–a greater awareness of one´s mortality’. Quotations presented have been selected to illustrate general features within each theme, facilitating a deeper and clearer understanding of participants experiences. Minority views are included as well and presented as ‘a few participants’ or similar.

### Concerns and worries in everyday life

The emotional responses to having been hospitalised and undergone a turbulent year of ups and downs on the path to recovery included not only fears about getting ill again but also worries about not being fully recovered as well as grief over lost abilities.

Participants generally felt anxious and concerned about their future. Although large steps towards recovery had taken place during the first months, a year afterwards participants felt that recovery had halted, and they were still more tired compared to before COVID-19.

I can´t tell what´s the hen and what´s the egg? If you feel tired and low on energy, you may get depressed, and when you´re depressed you feel tired. Sometimes I feel like…what the hell, there´s no use for this, is this even a life?. I can feel;is this a life? (worth living) *Woman 48 years*

Some participants felt that their self-esteem was lower; they felt more fragile, somewhat older or worried about getting ill again. It was mentioned that talking to others could make one feel better, whereas being at home with a lot of time for oneself and one’s worries made participants more anxious.

When you´re home and think a lot about things like that (worries about what could happen next)… these thoughts presents themselves involuntarily… and that´s why I try to keep busy and distracted. Sometimes when I’m sick, I feel like I’m going to die. Sometimes, I can actually feel that. *Man 59 years*

Worries about getting COVID-19 again prevented participants from going out or socialising as before. This was described as an ever-present fear in daily life, which participants always considered when planning daily activities. A few participants longed for contact with the healthcare system to receive information on how to handle their worries about the safety of going out.

For some participants, a generalised feeling of worry was now a part of their daily lives. Minor incidents that startled or surprised them, as well as information and impressions considered too emotional could make their hearts pound faster and it could be difficult to calm down. This heightened anxiety or feeling of panic affected in some cases participants’ ability to sleep. One participant asked himself every night if he dared to go to bed, while others woke up in the middle of the night, too anxious to go back to sleep.

When I’m about to go to bed, and I feel my heart racing… there´s no point to even try! (to sleep). My heart feels as if it’s going to jump out of my chest… A long time passed before I even dared to close my eyes. When I closed my eyes, these visions appeared, and I had these terrible nightmares. *Woman 62 years*

The fear of sleeping was also related to having listened to critically ill people fighting for their lives, or overhearing healthcare workers talking about fellow patients dying in the care unit. Some participants had difficulties forgetting the overall atmosphere of panic and stress that they had experienced in the care unit one year ago. However, all participants stressed that hospital healthcare workers had been highly professional, doing their best to make them feel as good as possible.

### Supportive and concerned relatives

Close family members and loved ones had meant a great deal during this first year of rehabilitation. Participants described how family members had given both emotional and practical support, and some explained that they would never have survived without them. Initially, the immediate family was described as being chocked and worried.

Those who visited me back then, they didn’t know what to believe really. They were used to see me as a strong and healthy person, and there I was; a tiny, curled up man sitting in a wheelchair! It came as a shock to see me. *Man 62 years*

Participants experienced closer bonds within the family due to an increased awareness about mortality and the transitory nature of life. It was evident that relatives had reflected on a life without their father, wife, husband or child in the aftermath of COVID-19. Apart from closer bonds, the illness also made some family members considerably more anxious. For example, participants described how parents called several times a day just to check that they were all right.

We text ‘goodnight’ and ‘goodmorning’ every day (laughs), but then I forgot ones, and of course she (mum) became worried. So then I said; we don’t need to be in touch every morning, it is sufficient to text in the evening. *Man 51 years*

Participants stated that they too felt excessively anxious that something would happen to their loved ones. Increased concerns about the immediate family were pronounced, for example, a fear of making one’s children orphans. This concern manifested itself in daily life as worries about getting sick again, being extra careful in everyday life, and being cautious when making life-changing decisions.

### A new way of life–sorrows and advantages

Some participants mourned their previous way of living and their lost personality. Not being able to socialise as before, to work-out as before or to have the same stamina at work anymore were common examples of what participants missed the most.

Really, I am not that social anymore. I used to organize things for my friends and family, but not anymore. I am usually delighted to enjoy others, but not anymore. Now, I have no energy to help out anymore, and it makes me feel bad. *Woman 62 years*

Some felt forced to lead their lives differently in order to cope, for example, by writing reminder notes, carrying out necessities only, or just working or resting a lot more during the daytime. There were examples of feeling shameful when not living up to other people’s expectations. Further, participants explained that lost functions, activities or lost personality traits also made them more emotional in everyday life. Some cried a few times a week and were easily moved to tears. For others it was important not to think about how they had changed or their new situation, in order to keep their spirits up.

I don’t think about the days when I was sick. I don’t dwell on the past. And I felt the same when I saw a psychologist… why should I dwell on this? There’s absolutely no point. Instead, I want to find ways to move forward. *Man 47 years*

Participants referred to their personalities when describing how they had approached life after COVID-19. Some said they did not like to dwell on negative life events, or that nothing good comes from dwelling on sorrows that have already happened. Others felt better after having cried, or when they could speak about the situation with a healthcare professional or a counsellor. Several longed for professional counselling, to help them combat strong emotional reactions after COVID-19.

I would really like to speak to someone who knows what to do… who knows how I’m supposed to think about this (my worries). I would like to know when I can expect to feel less anxious. *Man 59 years*

Participants described themselves as persistent when it came to finding and practising new altered ways of living. Several had come to the conclusion that they would never get back to life as it was before COVID-19. They reasoned that it was important to make the best of their new situation, accept lost capacities and to “play their new cards right”. Examples of alterations in life were going out fishing instead of working-out, planning a few days ahead instead of being spontaneous, spending more time with family instead of working long hours, or resting in between household chores instead of afterwards. A few participants tried to conceal their feelings of frustration, irritation and sadness from their relatives and did so on a daily basis.

It’s a great big sadness that I can’t work out anymore. Above all, this got to do with my fondness of working out, and that my body feels good to be trained. Me and my husband found a new interest that is not so physically demanding. We got a boat and learnt how to fish! *Woman 48 years*

### Seize the day—A greater awareness of one´s mortality

Participants described how they had gained different perspectives on life after COVID-19. Priorities had shifted from work and other duties to family life and unfulfilled dreams. Some said they wanted to work less and try to catch up with relatives whom they had not seen for a long time. Others claimed that they felt braver and more decisive, as if they no longer had anything to lose. When it came to new insights about life, there was a sense of gratitude for the personal growth that had taken place during the first year after COVID-19.

I cannot even imagine who I would have been if I hadn’t got sick (in COVID-19). Somehow I needed to go through all this…I wouldn’t have got the same insights without it. I have changed my personality somewhat, it hit me now, that I have changed. I am less scared I would say, and braver. *Man 51 years*

Participants wanted to take up new interests or to enjoy life to a greater extent after COVID-19. However, the shift in life focus was not always experienced as something joyous or positive; it was also described as a worry associated with not knowing what future life could hold in store, as well as a way to manage anxiety. Some said they should pull themselves together and do what they always longed to do, because one never knew whether misfortune might strike again. Others felt a great deal older after all that had happened during the past year and wanted to accomplish goals in life before it was too late.

A long time ago, my grandfather told me; don’t live your life as others say you should, live life the way you want to live it. And in the aftermath of COVID-19, I kind of get what he meant by this, and I live more on my terms now. We are more concerned to do what we both feel good to do, and get energy from. We want to take care of us, in a way that our bodies and minds will benefit from. *Woman 48 years*

Participants expressed in different ways how they wanted to take better care of their lives and make well-informed decisions one year after COVID-19. This changed approach to life was for some associated with a sense that life was more valuable now, whereas for others the changed attitude was related to the stress of not knowing what could happen next. This concern about the future was sometimes manifested in a pronounced reluctance to plan life in advance, for example, events or trips.

## Discussion

A year after hospitalisation due to COVID-19, it was evident that participants felt more anxious and worried in their daily lives compared to before the infection. The ever-present anxiety about becoming sick again strongly limited social contacts and daily routines to promote well-being. Previous research has found that staying home with one’s own thoughts and no longer carrying out appreciated activities may increase worries [16]. Community restrictions and pandemics can certainly lead to increased anxiety and unwanted behavioural changes [15], but it may be important to acknowledge when these anxiety responses become inadequate or abnormally high [13]. For example, when the risk of infection is low, it may be important to counteract negative emotional spirals by going out to meet other people and by trying to resume activities that provide pleasure [14, 17].

Results of the current study showed that participants stayed home to relieve anxieties rather than making new well-informed decisions about the risks involved in going out. Additionally, it is probable that the increased worries among close relatives may further have contributed to a rather closed family system with heightened anxiety [15]. In a sense, participants were stuck in their own forced lockdown, a lockdown guarded by anxiety [24].

The heightened anxiety made participants alert and easily frightened, with difficulties to calm down in everyday life. In line with this result, it has been shown that severe COVID-19, ICU experience and having witnessed other patients suffering during hospitalisation were risk factors for developing symptoms similar to those of PTSD [25]. In addition, our results indicate that even a year after their hospitalisation, people may need professional support to overcome emotionally disturbing symptoms [10]. It may also be important to help people release fears and anxieties that no longer serve their purpose when the imminent threat of COVID-19 is gone, with the goal to enable them to make realistic decisions in life again.

Our results showed that participants longed for professional counselling to help them cope with strong or difficult emotions after COVID-19. It is probable that this feeling of not receiving sufficient emotional support may increase anxieties on the path to recovery [17, 26]. It was previously shown that patients felt alone in their recovery after COVID-19 [26–28].

Participants expressed an identity loss accompanied by lower self-esteem and a sense of grief about not being able to recognise themselves anymore. Persisting symptoms after COVID-19 were found to impact people’s identity as healthy, independent or successful selves [17]. Additionally, previous findings show that consequences after COVID-19 may disrupt an individual’s professional self, relationships and overall sense of who one is [29].

It is likely that this feeling of having lost a part of one’s identity becomes manifest in a later phase after COVID-19, when people expect to have regained their prior lives [10]. In our study, long-term symptoms unavoidably affected participants’ ability to contribute, both professionally and in one’s personal sphere, which led to different degrees of experienced identity loss. In a previous qualitative study, participants felt guilt and shame over difficulties concerning their return to previous levels of function [19]. This result was partly confirmed in the present study, although participants in our study strongly emphasised the importance of finding and practising new altered ways of doing preferred activities in everyday life.

Participants clearly stressed that they still struggled with tiredness or fatigue one year after hospitalisation. It was recently found that nearly two-thirds reported fatigue at one year after ICU admission following COVID-19 [30]. The importance of recognising lost functions, not only as lost abilities but also as a loss of professional and individual identity, was raised in a recent British study [20]. It was argued that we need to acknowledge that people with long-term COVID-19 may experience challenging forms of identity loss which may lead them to feel more vulnerable [20]. Our results strengthen this conclusion, in that participants expressed a grief and sadness over lost functions, especially with regard to fatigue. This grief and confusion over not fully recognising oneself anymore may hinder processes of creating new positive and stable identities. Based on our results, it is likely that people need more professional support to understand their new reduced capacities and more support to create new positive identities.

A new approach to one’s life was clearly expressed by participants. This new approach was described as agonising, such as “how can I make the most of the time I have left to live?”; and also as a source of renewable joy, such as “I do more fun stuff now, you never know what could happen next”. Hence, an interesting dimension in these narratives was the mix of positive and negative emotions that had formed a re-evaluation of their outlook on life. On the one hand there were concerns and anxieties about future life, while on the other hand, participants felt fortunate to be alive, and life was seen as more valuable and precious.

It is well known that feelings of gratitude may develop after having been critically ill [31–33]. These subjectively experienced positive changes are referred to as posttraumatic growth (PTG), and in line with our results, PTG most often includes a more positive outlook on life and a greater appreciation of living [31, 34]. Participants in our study explained how their life priorities had shifted towards enhancing quality of life, i.e., from career-oriented to more family-oriented concerns. This supports the PTG theory that individual priorities shift towards reflecting more intrinsic, rather than extrinsic values [34]. Participants in the present study had to varying degrees changed their personalities and experienced PTG, an experience that of course differs from individual to individual [32]. For example, a previous finding showed that personal resources, e.g., coping strategies and social support, were positively associated with the development of PTG [33, 35].

However, while these new approaches and thoughts about life were evident a year after severe COVID-19, a question that naturally arises is to what extent these changes are constant. It remains unknown whether feelings of gratitude may represent a permanent personality change, or whether they will fade over time.

Even though men dominate in numbers among hospitalized patients after COVID-19, the low participation of women can be seen as a limitation of this study. Further research with a higher proportion of female participants, would help us understand more about women’s experiences of living with long-term consequences of COVID-19.

## Conclusions

A year after hospitalisation, participants described how they struggled to create a more tolerable life with more elements of joy and happiness in their new situation. Overall, the patients’ narratives were diverse, not only with regard to how participants described their coping in everyday life but also to what extent this struggle was experienced as difficult or emotionally challenging.

In the present study, participants most likely developed PTG as a result of experiencing a profound existential change, having encountered one’s mortality and survived. However, narratives also included grief, frustration over not recognising oneself, fatigue and tangible worries. As more time passes, perhaps without recovery or improved functions, it is difficult to know if feelings of grief and frustration will take over, or if PTG will be a permanent growth that will lead to acceptance. Further research can provide insights into more lasting consequences on mental ill-health for this group, potential for recurrence of common conditions (anxiety or depression), and strategies or interventions to support this group in a long-term perspective. Overall, it is highly important to address persons’ mental health needs a long time after severe COVID-19 to promote recovery, prevent long-term mental consequences, and provide support for rehabilitation.

## Supporting information

S1 File Interview guide in English. (DOCX)

S2 File Interview guide in Swedish. (DOCX)
