# Supplementary material for: Understanding concerns after severe COVID-19: A self-imposed lockdown guarded by anxiety?
Source: PLoS One. 2023 Jul 19;18(7):e0287981. doi: 10.1371/journal.pone.0287981 (PMC10355428; doi:10.1371/journal.pone.0287981)
Supplement: S1 File — (DOCX) [file pone.0287981.s001.docx]

# Twelve-month COVID-19 follow-up

# Aspects of patient health, rehabilitation, daily life and work: an interview study

- Objective: the interviews will explore patients’ perceptions of their overall well-being, mental health, rehabilitation and return to work.
- The plan is to gain insight into how the individuals feel and what their lives are like one year after their hospital stay for COVID-19.
- The interviewees will be informed about the project and its purpose. Does the interviewee have questions about its purpose?
- Signatures confirming their informed consent are required.

The questions will be based on the following areas, where you can describe your experience and perceptions today, roughly a year after your discharge from hospital after COVID-19 treatment:

- your personal health and mental well-being
- your current social life and scope for active community participation
- your work situation today and return to work after COVID-19.

## Health

How are you feeling today?

What symptoms of COVID-19 do you currently still have? (Focus on experience and/or symptoms that you think are clearly connected with your COVID-19 infection.)

## Daily life – activities and leisure

Do you feel that the symptoms related to COVID-19 affect you in your daily life? (How? Examples?)

Have your symptoms brought about any changes in your daily life (such as more rest or fewer daily activities)?

Do you have any other strategies for managing your symptoms in daily life? (Do you have strategies or other approaches to make your daily life easier?)

Have you changed your habits in terms of leisure activities and/or interests? (If so, how?)

Do you feel depressed or in lower spirits after your illness?

What is it like to fall ill with an infection that the health care system does not yet know much about?

## Social life – family and friends

Has your role in the family changed? (If so, how?)

Have your family relationships changed since you had COVID-19? (How?)

Have your relationships with friends and acquaintances, or how you socialise, changed because of your COVID-19 symptoms? (In what ways?)

Have you joined any face-to-face or social media groups of people with long COVID (long-term effects of COVID-19)?

## Working life

How much do you work now? (Percentage of full-time employment?)

Has your work capacity changed? If so, how?

Do you use any special strategies at work (if COVID-19 has affected your work capacity)?

Please describe your return to work after COVID-19.

Have you been on sick leave repeatedly — that is, tried to go back to work and then fallen ill again?

Please describe your work situation today.

How do you feel you have been treated at work since you returned?

Have you received the support you need?

What has been the key aspect of your return to work?

What are your expectations when it comes to your future working life?

If you have not yet returned to work, have you received any response from your workplace and/or employer? How would you describe that response?

## Changed approach

Has everything to do with the infection and hospital stay you have experienced changed your outlook on life? If so, how?

What are your thoughts about the future?

## Completion

Out of everything you have told us above, is there any particular information you feel is especially important for us to take away?

Is there anything else you would like to tell us?

## Supplementary questions

- Would you like to tell us more about it?
- In what way? What do you mean?
- Could you give an example of that kind of situation?
- I don’t really understand. What do you mean by “It’s connected”?
- How did you arrive at that view?
- How did you experience that? Or: How did you feel about that?
- How important is that to you?
- Remind the interviewee about the question topic.
