# Supplementary material for: Understanding concerns after severe COVID-19: A self-imposed lockdown guarded by anxiety?
Source: PLoS One. 2023 Jul 19;18(7):e0287981. doi: 10.1371/journal.pone.0287981 (PMC10355428; doi:10.1371/journal.pone.0287981)
Supplement: S2 File — (DOCX) [file pone.0287981.s002.docx]

# En 12-månaders uppföljning: Covid-19 – perspektiv kring hälsa, rehabilitering, vardagsliv och arbete för patienter: en intervjustudie

- Mål: Intervjuerna kommer att söka kunskap om patienters upplevelse kring sitt allmänna mående, sin psykiska hälsa, rehabilitering och arbetsåtergång.
- Tanken är att få en insyn i hur personerna mår och hur de lever ett år efter sjukhusvistelse för COVID-19
- Deltagaren informeras om projektet och projektets syfte. Har deltagaren frågor om projektets syfte?
- Informerat samtycke skrivs under.

Frågorna kommer att utgå från några områden, där du får beskriva dina erfarenheter och upplevelse idag ca tolv månader efter utskrivning från sjukhus på grund av vård för covid-19:

- Din egen hälsa och ditt psykiska mående.
- Ditt nuvarande sociala liv och din möjlighet att delta i samhället.
- Din arbetssituation idag samt din arbetsåtergång efter covid-19.

## Hälsa

Hur mår du idag?

Vilka symtom finns kvar nu efter covid-19? (koncentrera dig på upplevelse/symtom som du själv tänker har en tydlig koppling till din covid-19 infektion)

## Vardagsliv – sysslor och fritid

Upplever du att symtomen relaterat till covid-19 påverkar dig i vardagen? (på vilka sätt? Hur visar det sig?)

Har dina symptom medfört några förändringar i din vardag? (t.ex. vilar mer, färre aktiviteter per dag?)

Har du några andra strategier för att hantera dina symtom i vardagen? (har du strategier eller andra tillvägagångssätt för att underlätta i vardagen?)

Har du ändrat dina vanor när det gäller fritidsaktiviteter/intressen? (om ja, på vilka sätt)

Känner du dig deprimerad eller mer ledsen efter sjukdomen?

Hur är det att ha varit sjuk i en sjukdom som sjukvården ännu inte vet så mycket om?

## Socialt liv – familj och vänner

Har din roll i familjen ändrats? (om ja, på vilket sätt?)

Har dina relationer inom familjen förändrats efter covid? (på vilka sätt?)

Har dina relationer till vänner och bekanta, eller ditt sätt att umgås, förändrats som en följd av dina covid-19 symtom? (på vilka sätt?)

Har du gått med i någon grupp på sociala medier eller i verkligheten med andra som haft långtidsverkningar efter covid?

## Arbetsliv

I vilken omfattning arbetar du nu?

Har din arbetsförmåga ändrats? Isåfall hur?

Använder du dig av några speciella strategier i arbetet? (Om covid har påverkat arbetsförmågan)

Vill du beskriva din arbetsåtergång efter covid-19?

Har du varit sjukskriven i omgångar? D.v.s. försökt gå tillbaka i arbete och sedan återigen blivit sjukskriven?

Vill du beskriva din arbetssituation idag?

Hur upplever du att bemötandet på arbetsplatsen har varit sedan du kom tillbaka?

Har du fått det stöd som du behöver?

Vad har varit viktigast för din arbetsåtergång?

Hur ser dina förväntningar ut när det kommer till ditt fortsatta arbetsliv?

Om du inte är tillbaka i arbete än; har du fått någon respons från din arbetsplats/arbetsgivare? Hur skulle du vilja beskriva den responsen?

## Förändrat förhållningssätt

Har allt det här som du varit med om, infektionen och sjukhusvistelsen, har det förändrat din syn på livet, berätta i så fall hur?

Hur tänker du kring framtiden?

## Avslutning

Av allt som du har berättat om nu; är det något som du upplever som särskilt viktigt att vi får med oss?

Är det något ytterligare som du vill förmedla?
